# Dynamic Changes of Brain Cilia Transcriptomes across the Human Lifespan

**DOI:** 10.3390/ijms221910387

**Published:** 2021-09-27

**Authors:** Siwei Chen, Wedad Alhassen, Roudabeh Vakil Monfared, Benjamin Vachirakorntong, Surya M. Nauli, Pierre Baldi, Amal Alachkar

**Affiliations:** 1 Department of Computer Science, School of Information and Computer Sciences, University of California-Irvine, Irvine, CA 92617, USA; siweic@uci.edu (S.C.); pfbaldi@uci.edu (P.B.); 2 Institute for Genomics and Bioinformatics, School of Information and Computer Sciences, University of California-Irvine, Irvine, CA 92617, USA; 3 Department of Pharmaceutical Sciences, School of Pharmacy, University of California-Irvine, Irvine, CA 92617, USA; walhasse@uci.edu (W.A.); rvakilmo@uci.edu (R.V.M.); bvachira@uci.edu (B.V.); 4 Department of Biomedical and Pharmaceutical Sciences, School of Pharmacy, Chapman University Rinker Health Science Campus, Irvine, CA 92618, USA; nauli@chapman.edu

**Keywords:** cilia, human, brain, transcriptome, lifespan, linear regression

## Abstract

Almost all brain cells contain primary cilia, antennae-like microtubule sensory organelles, on their surface, which play critical roles in brain functions. During neurodevelopmental stages, cilia are essential for brain formation and maturation. In the adult brain, cilia play vital roles as signaling hubs that receive and transduce various signals and regulate cell-to-cell communications. These distinct roles suggest that cilia functions, and probably structures, change throughout the human lifespan. To further understand the age-dependent changes in cilia roles, we identified and analyzed age-dependent patterns of expression of cilia’s structural and functional components across the human lifespan. We acquired cilia transcriptomic data for 16 brain regions from the BrainSpan Atlas and analyzed the age-dependent expression patterns using a linear regression model by calculating the regression coefficient. We found that 67% of cilia transcripts were differentially expressed genes with age (DEGAs) in at least one brain region. The age-dependent expression was region-specific, with the highest and lowest numbers of DEGAs expressed in the ventrolateral prefrontal cortex and hippocampus, respectively. The majority of cilia DEGAs displayed upregulation with age in most of the brain regions. The transcripts encoding cilia basal body components formed the majority of cilia DEGAs, and adjacent cerebral cortices exhibited large overlapping pairs of cilia DEGAs. Most remarkably, specific α/β-tubulin subunits (*TUBA1A*, *TUBB2A*, and *TUBB2B)* and *SNAP-25* exhibited the highest rates of downregulation and upregulation, respectively, across age in almost all brain regions. α/β-tubulins and *SNAP-25* expressions are known to be dysregulated in age-related neurodevelopmental and neurodegenerative disorders. Our results support a role for the high dynamics of cilia structural and functional components across the lifespan in the normal physiology of brain circuits. Furthermore, they suggest a crucial role for cilia signaling in the pathophysiological mechanisms of age-related psychiatric/neurological disorders.

## 1. Introduction

Primary cilia, antennae-like microtubule sensory organelles, protrude from the membranes of almost all cell types. Once considered vestigial organelles, brain cilia are critical for brain development [1,2,3,4,5,6]. The crucial roles for cilia in neurogenesis, neuronal differentiation, and maturation have been well established, and cilia dysfunctions during the embryonic stages have been associated with abnormalities in the brain’s formation, morphology, and connectivity [7,8,9,10]. Further, most primary cilia dysfunctions (called ciliopathies) are associated with neurological disorders, including cognitive deficits [11,12,13]. We have recently uncovered evidence for the dysregulation of cilia’s structural and functional components in the neurodevelopmental disorders schizophrenia and autism [13].

Beyond the developmental stage, cilia are continuously required for proper functions of the mature brain [5,14,15]. Besides their essential role in continuous neurogenesis in two regions of the adult brain (hippocampal dentate gyrus and ventricular–subventricular zone) [14], cilia act as signaling hubs to receive and transduce various signals and regulate cell–cell communications (see [15,16] for reviews).

Cilia’s highly specialized sensory and transducing functions during developmental and mature stages are mediated by distinctive substructural and functional components, including basal body, axoneme, centrosome, intraflagellar transport (IFT) machinery, and cell membrane receptors [17,18,19,20,21,22]. The distinct roles of brain cilia during developmental and mature stages trigger the question of whether cilia morphologies and structures change across human lifespan [7,8,9,10,14]. 

We have recently demonstrated a high degree of circadian rhythmicity of cilia genes’ expression across primate brain areas [23]. We also demonstrated that cilia length is highly dynamic in response to many internal and external stimulations, including genetic, optogenetic, chemogenetic, and chemical manipulations [24]. However, the age-dependent dynamics of cilia substructural and functional components are not known. Further, whether cilia age-dependent dynamics have any role in brain functions and in age-related pathological conditions such as neurodevelopmental and neurodegenerative disorders have not yet been explored. We hypothesize here that genes encoding cilia structural and functional components responsible for driving cilia regulation of neuronal generation, growth, differentiation, and communications, are highly dynamic and are expressed in age-dependent patterns in developing and adult brains. To test this hypothesis, we acquired transcriptomic data from the BrainSpan Atlas of the Developing Human Brain web portal under the developmental transcriptome dataset to profile age-dependent expressions of brain cilia genes [25]. We then computed the goodness of fitting the age-dependent expression into linear regression models to define age-dependent changes in cilia gene expression patterns. 

## 2. Results

### 2.1. Cilia Transcripts Exhibit High Age-Dependent Expression

**Figure 1:**
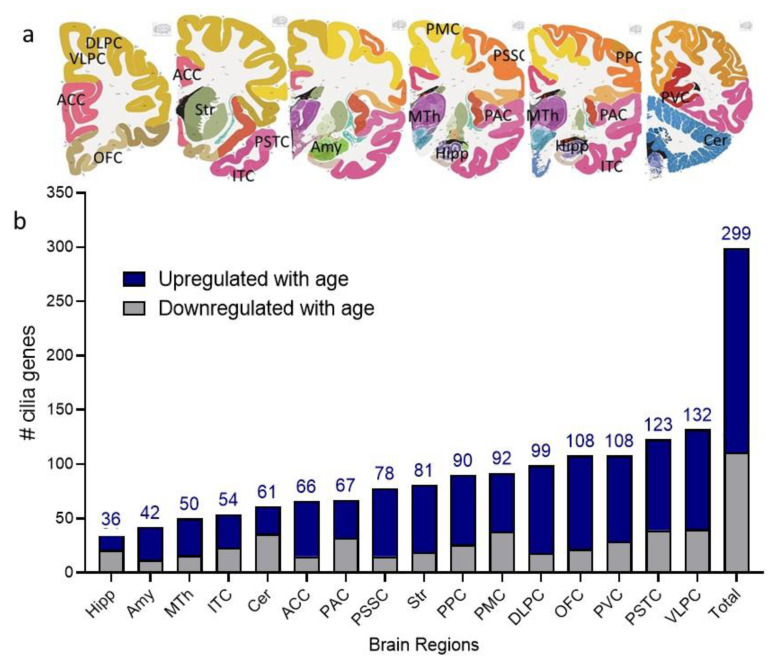
Age-dependent gene expressions of cilia genes abundance in the human brain. (**a**) Representative brain section from human brains (BrainMaps: An Interactive Multiresolution Brain Atlas; http://brainmaps.org [retrieved on 2 August 2021] [26] showing the regions and nuclei from which tissues were collected: anterior (rostral) cingulate (medial prefrontal) cortex (ACC), amygdaloid (Amy), Cerebellar cortex (Cer), dorsolateral prefrontal cortex (DLPC), Hippocampus (Hipp), Inferolateral temporal cortex (area TEv, area 20) (ITC), Mediodorsal nucleus of the thalamus (MTh), Orbital frontal cortex (OFC), posterior (caudal) superior temporal cortex (area 22c) (PSTC), posteroventral (inferior) parietal cortex (PPC), primary auditory cortex (core) (PAC), primary motor cortex (area M1, area 4) (PMC), primary visual cortex (striate cortex, area V117) (PVC), primary somatosensory cortex (area S1, areas 3,1,2) (PSSC), Striatum (Str), and ventrolateral prefrontal cortex (VLPC).(**b**) Stacked bar chart shows the total number of cilia genes that are differentially expressed with age (cilia DEGAs) in the whole brain and each of the 16 brain regions, with the exact number of cilia DEGAs that are upregulated and downregulated. The numbers on the top of bars represent the number of DEGAs in brain regions.

To investigate how cilia genes’ expressions change with age, we enquired RNA-Seq data from the BrainSpan Atlas of the Developing Human Brain. Sixteen brain regions reported in the BrainSpan Atlas of the Developing Human Brain [25] had complete chronological age-related cilia transcripts data (Table S1) (https://www.brainspan.org/static/download.html) [Retrieved on 10 April 2021]. Using linear regression to model the gene expression level as a function of the age, we found that out of 445 cilia transcripts, 299 (67% of cilia transcripts) were determined to follow linear regression in at least one brain region (regression coefficient at *p* < 0.05, Figure 1b). The age-dependent expression was region-specific, with the VLPC exhibiting the highest number of cilia DEGAs (132 cilia DEGAs) followed by PSTC (123 cilia DEGAs), PVC and OFC (108 cilia DEGAs in each), and DLPC (99 cilia DEGAs), primary PMC (91 cilia DEGAs), and PPC (90 cilia DEGAs) (Figure 1b). Hippocampus and amygdala exhibited the lowest number of cilia age-regulated transcripts (36 and 42 cilia age-regulated transcripts in the hippocampus and amygdala, respectively) (Figure 1b). The majority (188 genes) of the cilia DEGAs followed upregulation patterns with age (Figure 1b). In any given brain region, except for the hippocampus and cerebellum, the majority of cilia DEGAs exhibited increased expression with age progress (Figure 1b). 

**Figure 2:**
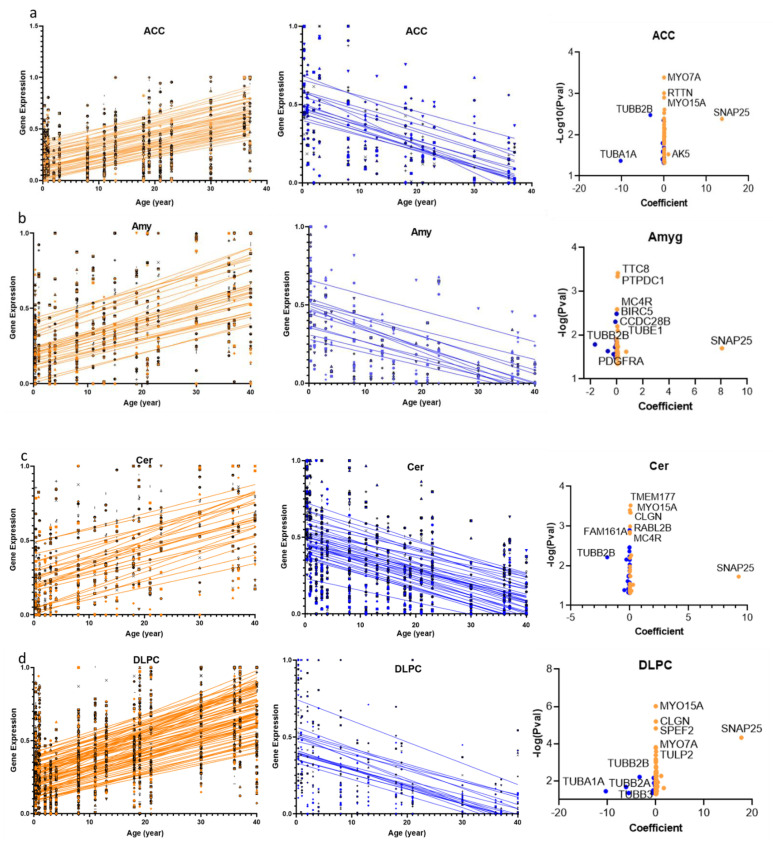

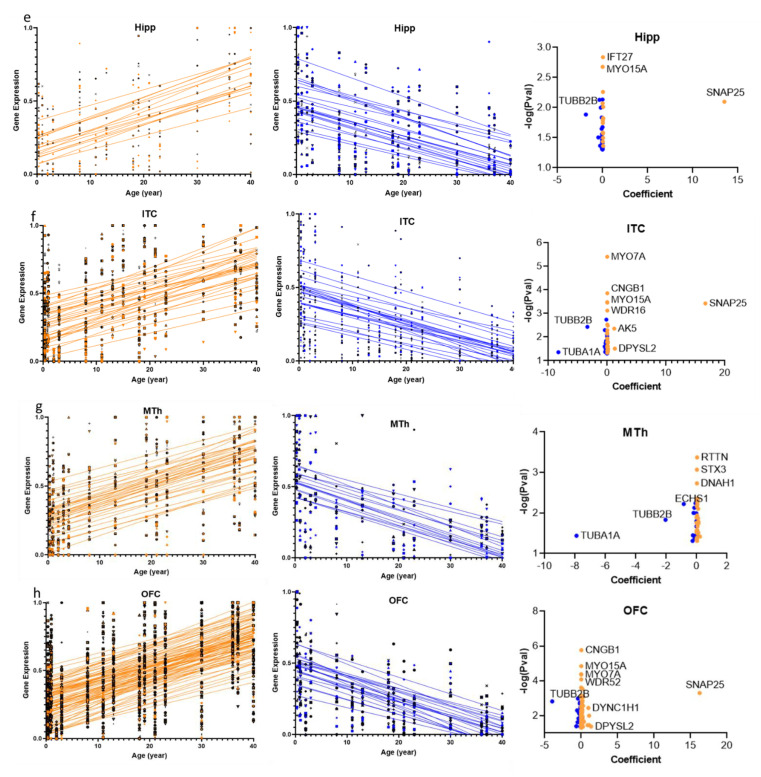

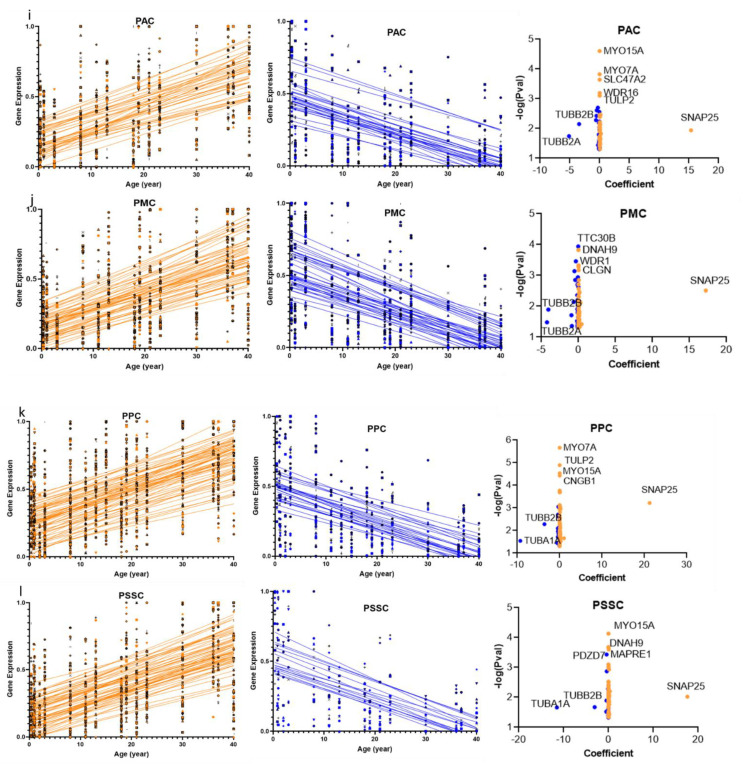

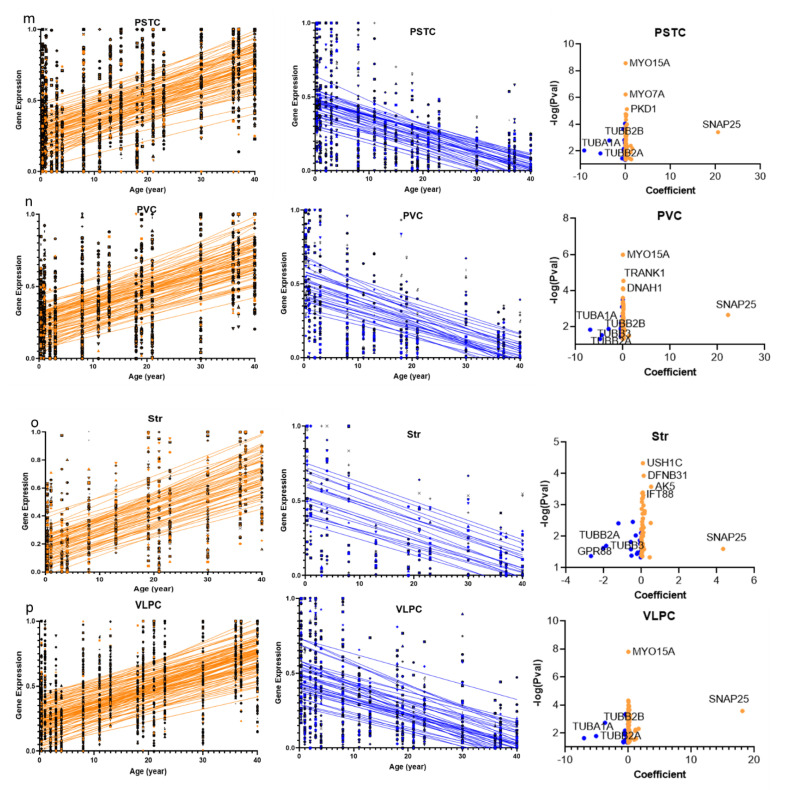
Linear regression model fitting the effect of age on cilia gene expression in 16 human brain regions. (**a**–**p**): scatter plots of gene expression patterns of cilia genes that are differentially expressed with age (cilia DEGAs) in each of the 16 human brain regions. (**Left panels**): Age-dependent upregulated cilia DEGAs in brain regions. (**Middle panels**): Age-dependent downregulated cilia DEGAs in brain regions. (**Right panels**): Volcano plot of regression coefficients and *p*-values in brain regions. The x-axis is the age effect on cilia gene expression of cilia DEGAs measured by regression coefficient. The y-axis represents the −log10 (*p*-value). The larger −log10 (*p*-value) value, the more significance. The orange dots represent age-dependent upregulated cilia DEGAs and the blue dots represent age-dependent downregulated cilia DEGAs.

Among all cilia DEGAs, *SNAP-25* exhibited the highest linear regression coefficient compared to other cilia genes in any given brain region, with the highest regression coefficient value in PVC (22.3) (Figure 2a–p, Table S2). Around a four-fold increase in *SNAP-25* expression in the PVC between the first 5-year age group (0–5 years) and the last age group (36–40). On the other hand, genes encoding α-tubulin subunit (*TUBA1A*) and β-tubulin subunits (*TUBB2A and TUBB2B*) exhibited the top negative coefficients, with a region-specific distribution of either one or more of these tubulin subunits. For example, in the thalamus and most cortical regions, both *TUBA1A* and *TUBB2A/TUBB2B* exhibited the highest negative slopes among cilia DEGAs, with *TUBA1A* displaying a higher absolute coefficient than *TUBB2A/TUBB2B* in these regions (Figure 2a–p, Table S2). Genes encoding β-tubulin subunits TUBB2A/TUBB2B exhibited the highest negative coefficient in brain regions, whereas *TUBA1A* was not DEGA gene. These regions included OFC and non-cortical regions, with either *TUBB2B* or *TUBB2A* being the DEGA in a given region. Thus, *TUBB2B* exhibited the highest negative coefficient in the OFC: −4.0, whereas *TUBB2A* exhibited the highest negative coefficient in the Str: −2.0. In addition, in the cortical regions PAC and PMC, both *TUBB2A* and *TUBB2B* were the top downregulated DEGAs, with regression coefficients for *TUBB2A/TUBB2B* as of −5.1/−3.4 and −4.2/−4.1 in the PAC and PMC, respectively (Figure 2a–p, Table S2).

### 2.2. Overlap of the Expressions of Age-Regulated Cilia Transcripts across Brain Regions

We computed the Pearson correlation coefficient (R) for every pair of age-regulated genes. We generated a correlation matrix based on the correlation of gene expression levels among all age-dependent genes in each region (Figure S1a–p). Most of the cilia DEGAs that were upregulated with chronological age clustered closely together, and cilia DEGAs that followed negative slope clustered together (Figure S1a–p). 

**Figure 3:**
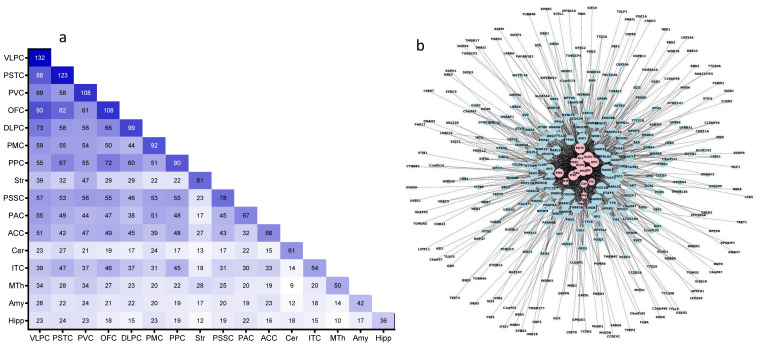
Overlap of cilia DEGAs transcriptome in brain nuclei/regions. (**a**) Heatmap of region-by-region intersections of the cilia DEGAs genes. The size of the intersections ranged from 9 (light blue) to 93 transcripts (dark blue), which occurred in two intersections. (**b**) Network graph represents the cilia DEGAs-region interaction, using “spring” layout from the NetworkX package with Python. Each blue node presents one of the cilia DEGAs, and each red node presents the brain. Edges were drawn to connect cilia DEGAs nodes to brain regions nodes where they are expressed. The node’s size is proportional to its connectedness in the graph: the more connected the node is, the bigger its size.

A wide expression overlap of age-regulated cilia transcripts among brain regions was observed (Figure 3a,b). Only 93 cilia transcripts exhibited a unique age-regulated pattern in a single brain region (Figure 3a,b). We screened cilia transcripts that exhibited age-dependent expressions in multiple brain regions and found that *CCDC28B* was the only cilia DEGA common across all brain regions. However, CCDC28B age-dependent expression patterns differed across brain regions [27,28], being downregulated in 12 and upregulated in four brain regions. 

Other cilia DEGAs shared between multiple brain regions included *TUBB2B*, *MYO15A*, *SNAP25*, and *TRANK1* shared in 15 brain regions, *MAPRE1*, *TULP2*, and *FBLN2* shared in 14 brain regions (Figure 3a,b). 

Regarding region view overlap, all brain regions exhibited an overlap of their cilia DEGAs with other brain regions. The number of overlapped DEGAs among brain regions ranged from 93 genes (between VLPC and OFC) to nine genes (between MTh and Cer) (Figure 3a,b).

### 2.3. Structural and Functional Organization of Age-Regulated Cilia Genes

**Figure 4:**
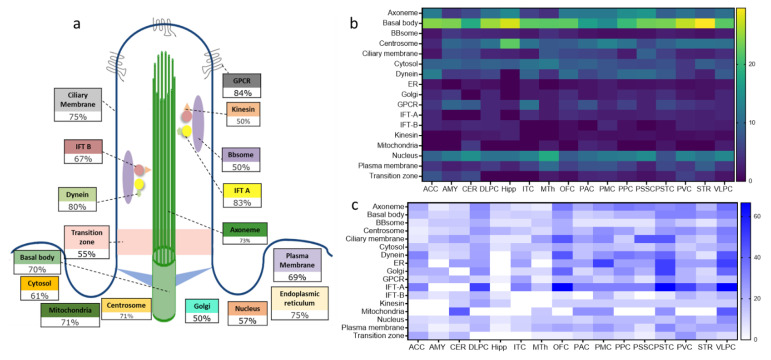
Sub-structural organization of cilia DEGAs genes. (**a**) Percentages of the total DEGAs genes in each of the cilia sub-structural compartments as proportions to the total cilia genes in each sub-structural compartments. (**b**) Heatmap of cilia sub-structural DEGAs presented as proportions to the total cilia transcripts in 16 brain regions. Yellow indicates a high percentage of DEGAs genes, and dark blue indicates no DEGAs genes in a given brain region. (**c**) Heatmap of the cilia sub-structural DEGAs presented as proportions to the total corresponding sub-structural transcripts in 16 brain regions. Dark blue indicates a high percentage of upregulated genes, and white indicates no DEGAs in a given brain region.

We then examined the distribution of age-regulated cilia genes across cilia’s main structural and functional components (Figure 4a). We found that the total number of transcripts encoding the basal body components in the 16 brain areas exhibited the highest number (63) of DEGAs (70% of total basal body transcripts). In contrast, BBSomes, kinesin, and Golgi components exhibited minor age-dependent changes of expression (50% of their corresponding total transcripts) (Figure 4a). Strikingly, 84% of total transcripts encoding the components of the GPCRs exhibited age-dependent expressions (21 GPCRs), whereas 83.3%, 66.7%, 54.6%, 75%, 73.0%, and 80% of IFT-A, IFT-B, transition zone, cilia membrane, axoneme, and dynein, respectively exhibited were DEGAs in at least one brain region (Figure 4a). This finding strongly supports the age-dependent regulation of cilia assembly and functions.

Although the abundance of DEGAs of cilia substructural and functional components varied across brain regions, the age-dependent changes in expression of the cilia sub-structural and functional components were primarily consistent with their age-dependent patterns in the whole brain (Figure 4b and Figure S1a–p). Thus, genes encoding the basal body exhibited the highest numbers of DEGAs in all the 16 brain regions, with a percentage of basal body DEGAs ranging from 14% to 30% of total basal body transcripts in the PMC and Str, respectively. However, DEGAs of other cilia substructures’ components varied widely across brain regions. For example, while DEGAs formed 66% of genes encoding IFT-A particles’ components in the OFC, PSTC, and VLPC, none of the IFT-A genes in the Amy, Cer, and Hipp were DEGAs (Figure 4c and Figure S1a–p). Similarly, the percentage of genes encoding dynein motor particles that were DEGAs varied from 0% in the hippocampus to 44% in the OFC and VLPC (Figure 4c and Figure S1a–p). The patterns of age-dependent changes of gene expression of cilia substructural and functional components were also region-dependent. For example, while, most cilia DEGAs apparently increased in expression with age, 100% of the cilia-associated mitochondrial DEGAs were downregulated with age in all brain regions except the cerebellum (Figure S2a–p). A total of 100% of the BBSomes DEGAs were upregulated in 15 brain regions, whereas the single BBSomes DEGA in the PMC was downregulated with age (Figure S2a–p). 

## 3. Discussion

In this study, we examined whether the expressions of genes encoding cilia structural and functional components change in age-dependent manners, and we determined the patterns of the changes. To evaluate the age-dependent changes in cilia genes’ expressions in the human brain, we acquired publicly available transcriptomic data of various brain areas from the BrainSpan Atlas of the Developing Human Brain [25]. We computed the goodness of fitting a linear regression to model the age-dependent changes in cilia gene expression by calculating the linear regression coefficient.

### 3.1. Cilia Transcripts Are Dynamic, and Their Age-Regulated Expressions Are Region-Specific 

The majority of cilia transcripts exhibited age-dependent changes in expression across at least one of the 16 brain regions reported in the human atlas [25]. In almost all brain regions, the majority of cilia age-regulated transcripts followed positive slopes of linear regression with age progress. The age-dependent alterations of transcript seem to be region-specific. In general, cerebral cortices (VLPC, PSTC, OFC, PVC, DLPC, PMC, and PPC) exhibited higher cilia age-dependent dynamics than the cerebellum and subcortical regions (hippocampus, thalamus, and striatum). Remarkably, cilia DEGAs exhibited higher overlap in anatomically adjacent or functionally connected brain regions. For example, the three frontal cortex sub-areas VLPC, OFC, and DLPC shared the largest cilia DEGAs. In contrast, the cerebellum and hippocampus shared the least cilia DEGAs with other brains regions. Similarly, the PSTC shared more cilia DEGAs with the anatomically adjacent frontal cortex regions (VLPC, OFC, and DLPC) than non-adjacent regions (ITC). This finding may indicate distinct roles for cilia in different brain regions and that functionally and adjacent regions may share cilia function at a specific age. We have previously shown that the cerebellum exhibits the lowest overlap of cilia rhythmic transcripts with other brain regions [23]. Therefore, the low overlaps of cilia dynamic transcripts between the cerebellum and other brain regions are not surprising as it reflects the distinct anatomical and functional organization of the cerebellum vs. cerebrum. 

The hippocampus exhibited the lowest number of cilia DEGAs. Unlike other brain regions, most of its cilia DEGAs followed a negative slope with age (more genes are downregulated with age). The hippocampal dentate gyrus is one of only two brain regions with ongoing neurogenesis in the postnatal and adult brain [14]. Thus, the low dynamics of cilia transcripts’ across age suggests comparable needs for cilia in the neurogenesis process in the hippocampus during human brain development and maturation. The fact that hippocampal neurogenesis decreases with age [29] and the tendency of cilia DEGAs to be downregulated with age (negative regression coefficient) in this region, our results might suggest that cilia’ role in the neurogenesis regulation is age-dependent. 

Not surprisingly, though interestingly, the brain regions that exhibited a low age-dependent change of cilia genes (the amygdala, the hippocampus, and the cerebellum) also exhibited low 24 h circadian oscillation [23]. These observations indicate that cilia structures and functions in these three brain regions are less dynamic across 24 h and lifespan than other brain regions. 

### 3.2. Distinct Age-Dependent Dynamics of Cilia Structural and Functional Components 

The core structure of primary cilia comprises a 9 + 0 arrangement of microtubules called axoneme, which extends from the cilia basal body to their tip through the ciliary transition zone [30,31]. The most considerable fraction of cilia DEGAs in the 16 brain regions were genes encoding for the basal body and axoneme components. The observation that the majority of DEGAs of axoneme, basal body, and transition zones were upregulated with age triggers the question of whether cilia length and morphology follow similar patterns of changes across the lifespan. A recent study reported age-dependent alterations in the number of ciliated neurons and cilia lengths in specific brain regions [32]. However, it is unknown whether similar changes in cilia length and numbers occur in other brain regions. 

Microtubules are formed of polymerized heterodimers of α-tubulin and β-tubulin. In many brain regions, specific genes encoding α-tubulins (mainly *TUBA1A*, and rarely *TUBA1C*) and β-tubulins (*TUBB2A*, *TUBB2B*, and *TUBB3*) were dynamic, with a consistent tendency to decrease in expression with age. Strikingly, these DEGAs tubulins, which form the basic building blocks of basal body microtubules, exhibited the topmost negative coefficient values compared to all other cilia DEGAs. *TUBA1A* represents the primary subunit gene linked to a group of tubulin disorders called “tubulinopathies” [33,34]. Other “tubulinopathies” genes include *TUBA1A*, *TUBB2A*, *TUBB2B*, *TUBA8*, *TUBB3*, *TUBB*, and *TUBG1* [35,36,37,38,39,40], which were found to be DEGAs in various brain regions. Tubulinopathies are characterized by various clinical features and are accompanied by a broad spectrum of malformations of cerebral cortices and subcortical structures, including the corpus callosum, the brainstem, and the basal ganglia [41]. Loss of α-tubulin and β-tubulin is a hallmark of chronic alcohol consumption and natural brain aging [42]. Along with these reports, our observations trigger the question of whether accelerated age-dependent loss of these cilia tubulins may contribute to the pathophysiological mechanisms of tubulinopathies. 

Unlike α-tubulin and β-tubulin, γ-tubulin is not a component of cilia axonemal microtubule, but it is a constituent of microtubule-organizing centers (MTOCs) including all non-centrosomal MTOCs [43,44,45,46]. However, γ-tubulin is a key player in the nucleation of microtubule assembly and in the formation of microtubule polarity [47,48,49]. The absence of functional γ-tubulin in the non-centrosomal MTOCs results in MTOCs loss or failure to nucleate and organize microtubules, which may cause lethality early in development [50]. Contrary to the age-dependent downregulation of α/β-tubulin DEGAs, genes encoding γ-tubulin complex associated proteins (*TUBGCP3*, *TUBGCP4*, *TUBGCP5*, and *TUBGCP6*) were upregulated with chronological age in multiple brain regions. This is surprising, given that γ-tubulin complexes are crucial to forming the templates for the longitudinal association with α-tubulin and β-tubulin dimers [51]. Hence, one would expect that the age-dependent expressions of γ-tubulin should be positively correlated with those of α-tubulin and β-tubulin. 

*SNAP-25* (Synaptosome Associated Protein 25) is another basal body component that exhibited a significant age-dependent change in expression. *SNAP25* gene expression increased with age in 14 brain regions, exhibiting the highest positive linear regression coefficient compared to all other cilia DEGAs and reaching 22.3 in the PVC. Indeed, SNAP25 was the only cilia dynamic DEGA with a positive coefficient value of >2 in any of the 16 regions. 

*SNAP-25* gene has been associated with several human psychiatric disorders, including attention-deficit/hyperactivity disorder (ADHD), schizophrenia, and bipolar disorder [52,53,54,55,56,57,58,59,60,61]. Interestingly, postmortem studies on schizophrenia patients’ brains have shown that altered protein levels of *SNAP-25* are specific to regions of the brain [55,62]. For example, SNAP-25 protein expression is reduced in the hippocampus, and anterior prefrontal cortex, whereas its expression is increased in the cingulate cortex and dorsolateral and medial prefrontal cortex (Broadman’s area 9) of schizophrenia patients. The variability of SNAP-25 protein levels in different brain areas has been proposed to contribute to the manifestations of variable behavioral symptoms in some schizophrenia patients [63,64,65,66,67,68]. 

Further, *SNAP-25* single nucleotide polymorphisms (SNPs) have been associated with distinct behavioral traits in children with ASD. For example, SNP rs363039 has been associated with cognitive deficits, whereas SNP rs363043 has been associated with hyperactivity traits [64,65,69,70,71]. 

Given that the *SNAP-25a* is the most abundant isoform of *SNAP-25* during the early weeks of life whereas *SNAP-25b* isoform’s expression becomes abundant in the adult brain [72,73], our finding of the striking increase in *SNAP-25* with age may reflect such age-dependent abundance of isoforms *SNAP-25a* in the developmental stage and *SNAP-25b* in adulthood [73,74]. Increased levels of *SNAP-25b* isoform impair synaptic transmission and maturation, and higher *SNAP-25b* expressions in the prefrontal cortex correlate with early-onset bipolar disorder during adolescence, which is considered more closely related to schizophrenia than to bipolar disorder itself [75]. 

Besides its crucial role in developmental disorders, *SNAP-25* is involved in aging-related disorders such as Alzheimer’s disease. For example, both *SNAP-25* SNPs rs363,043 and rs363,050 are associated with Alzheimer’s disease, and *SNAP-25* expression levels are decreased in multiple regions of the brains of Alzheimer’s patients [76,77]. Thus, our finding of the remarkable age-dependent increase in *SNAP-25* expression across the lifespan is of particular significance in this context and may suggest the need for finely balanced levels of *SNAP-25* expressions across different brain regions at every stage of the human lifespan. 

*CCDC28B* was the only cilia DEGA common across all brain regions, though it showed a different change trend across age in different brain regions. *CCDC28B* encoded protein is Coiled-Coil Domain Containing 28B, one of Bardet–Biedl syndrome-related proteins. *CCDC28B* protein localizes to centrosomes and basal bodies, wherein it plays a crucial role in ciliogenesis and cilia length regulation [27,28] through its interaction with kinesin-1 molecular motor [78]. 

Intraflagellar transport (IFT) machinery is essential for cilia genesis and maintenance [79]. With IFT sub-complexes IFT-A and IFT-B and IFT-associated proteins (BBSomes), kinesin and dynein motors perform the bidirectional transport process [80,81]. Kinesin-dependent motors and IFT-B particles are responsible for anterograde transports of cilia cargo towards the cilia tip. In contrast, dynein-dependent motors and IF-A particles are responsible for the retrograde transport from the cilia tip to their base [80,81,82]. The only kinesin DEGA was *KIF17*, which exhibited consistent upregulation with age in almost all brain regions, indicating that the kinesin motors functions are age-independent. While a few genes encoding IFT-A (*WDR19* and *IFT172*) and IFT-B (*IFT88*, *IFT80*, *TTC30B*, and *TTC30A*) changed in expression with age, BBSomes were less dynamic than other cilia components, and the vast majority of the BBSomes DEGAs exhibited an increase in expression with age. 

Remarkably, most genes that encode centrosome components displayed downregulation with age in multiple brain regions. The centrosome is the microtubule-organizing center, and its activity is critical for many cellular and developmental functions, particularly neurogenesis. Thus, the negative slope of the age-dependent expression changes of centrosome components may reflect the limited neurogenesis and proliferation in the adult brain. The only two components that consistently exhibited upregulation with chronological age were *RABL2A* and *RABL2B*, members of a RAB family of GTPases. The centrosomes provide a matrix of primary cilia. Mutations of centrosome genes produce structural and functional changes in primary cilia and result in a spectrum of clinical outcomes, including intellectual disability [83,84,85].

The cilia-associated GPCRs play vital roles in regulating cilia formation and structure and cilia’ ability to respond to external stimuli. Evidently, a significant part of ciliary signal transduction is mediated by GPCRs localized on the cilia membrane. 

Cilia DEGA-GPCRs included receptors that bind to identified ligands (neurotransmitters, neuropeptides, hormone) (*MCHR1*, *MC4R*, *GALR3*, *GALR2*, *DRD2*, *DRD5*, *KISSR1*, *SMO*, *SSTR3*, *NPY2R*, *NPY5R*, *LPAR6*, *NMUR1*, and *HTR6*) as well as orphan receptors (GPR88, *GPR161*, *GPR83*) [21,86,87,88,89]. Signaling through cilia GPCRs regulates a broad spectrum of physiological functions at different life stages, including feeding behavior, sleep, learning and memory, movement control, and reproduction [20,23]. Thus, the high degree of age-dependent dynamics of cilia GPCRs is conceivable given various functions throughout the human lifespan. 

Of particular interest is the decrease with age of the GPCR smoothened (*SMO*) expression in five brain regions, including the cerebellum and four cerebral cortices (PAC, PMC, PPC, VLPC). SMO transduces the activation of hedgehog (Hh) signaling that drives proliferation and differentiation during development, and its proper functionality is crucial for proper morphogenesis during embryonic development. Thus, *SMO* downregulation with age is not surprising and may reflect the age-dependent decreased need for SMO-Hh signaling pathway activation.

Remarkably, most cilia-associated GPCRs that exhibited age-dependent expression were found to follow circadian rhythms [23]. In addition, we previously showed striking alterations in cilia GPCRs’ expressions in neurodevelopmental and psychiatric disorders such as schizophrenia, in which 75% of cilia GPCRs are dysregulated [13]. Thus, the high 24 h rhythmicity and age-dependent dynamics of cilia-associated GPCRs indicate that their expressions are finely tuned across 24 h time and lifespan. Furthermore, GPCRs are highly druggable targets, with around 30–40% of drugs available in the market and the vast majority of therapeutic strategies for psychiatric disorders targeting GPCRs. Therefore, the developments of cilia-associated GPCRs targeted drug delivery might lead to novel treatments or prevention strategies for cilia-associated psychiatric and neurological disorders, should the treatments be administered at the proper age. 

## 4. Methods and Experimental Designs

### 4.1. Cilia Genes’ List 

Our cilia gene list was generated as previously reported [13]. Briefly, genes involved in cilia function were probed from databases including SysCilia Gold Standard (SCGS) version 1 database, CilDB database, and CiliaCarta [90,91,92,93]. Our search criteria included *Homo sapiens* species, gold standard cilia genes, and gene ontology annotated genes. Further, we included GPCRs known to localize on cilia from Schou et al. [88]. Next, cilia genes were verified by the CilDB database. Finally, cilia gene location was obtained from SysCilia and confirmed using the human protein atlas. Using these datasets, we identified and verified a list of 445 cilia genes (Table S1). We disclose that these cilia genes are a working list and are continuously being updated. It will never be ‘complete’ as new cilia genes will continue to be identified.

### 4.2. Brain Lifespan’ Gene Expression 

Cilia genes’ RNA-Seq data were obtained from the BrainSpan Atlas of the Developing Human Brain web portal under the developmental transcriptome dataset (https://www.brainspan.org/static/download.html) [Retrieved on 10 April 2021] [25]. Datasets include 42 individual brain samples from 16 different brain regions of 19 females and 23 males ranging from 4 months to 40 years old, to cover early infancy, early childhood, late childhood, adolescence, and adulthood stages. The brain regions included the amygdaloid (Amy), anterior (rostral) cingulate (medial prefrontal) cortex (ACC), cerebellar cortex (Cer), dorsolateral prefrontal cortex (DLPC), hippocampus (Hipp), inferolateral cortex (ITC), mediodorsal nucleus of the thalamus (MTh), orbital frontal cortex (OFC), posterior superior temporal cortex (PSTC), posteroventral parietal cortex (PPC), primary auditory cortex (PAC), primary motor cortex (PMC), primary visual cortex (PVC), primary somatosensory cortex (PSSC), striatum (Str), and ventrolateral prefrontal cortex (VLPC). 

### 4.3. Fitting the Data into a Linear Regression Model

The most straightforward approach for biological age modeling relies on linear regression of measured parameters, gene expression in our case, on chronological age. For each brain region and each gene, we used linear regression to model the gene expression level as a function of age. Age was treated as a continuous variable expressed in years, ranging from 0.33 (4 months) to 40. Next, we selected the linearly associated genes with age in at least one brain region for further investigation. These genes were referred to as cilia Differentially Expressed Genes with Age (cilia DEGAs). For each one of the 445 cilia genes and each of the 16 brain regions, we conducted a linear regression (LR) model between the independent variable (age) and the dependent variable (gene expression level). We obtained a corresponding *p*-value for each LR model. To adjust for multiple hypothesis testing, we computed the corresponding q values or False Discovery Rates (FDRs) (Table S2). However, we used a cutoff of 0.05 on the raw *p*-values to narrow down our search from 7120 (=445 × 16) gene-region pairs to 1288 gene-region pairs with strong evidence for a linear trend. Then, within the 1288 gene–region pairs, we used multiple lines of evidence to draw any conclusions. We primarily focused on genes with FDR-corrected values below 0.05. However, we also considered other factors such as the slope of the regression lines (positive vs. negative, large vs. small) and the overlap between regions, i.e., genes whose expression is linearly associated with age across multiple brain regions. 

To compute the power of the test, we used the GPower tool (version 3.1.9.6) and Statistical power calculator https://www.statskingdom.com/33test_power_regression.html, [accessed on 10 September 2021], and we chose a type I error of 0.05.

We computed the Pearson correlation coefficient (R) for every pair of age-regulated genes. In addition, we generated a correlation matrix based on the correlation of gene expression levels among all age-dependent genes in each region. 

Cilia DEGAs were clustered according to their localization to the cilium and functions, including axoneme, basal body, transition zone, kinesin, dynein, IFTA, IFTB, BBSome, Golgi, cytosol, nucleus, and the ciliary membrane. For sub-structural components with unknown localization, the genes were clustered in the “other” group. The percentages of sub-structural cilia transcripts that exhibited age-dependent expression were calculated.

## 5. Conclusions

In line with our previous findings, which demonstrated the high circadian oscillations of cilia genes across 24 h, our current results reveal a high dynamics of cilia structural and functional components across the lifespan. Our results suggest fundamentally crucial roles for ciliary signaling in the functions of the developing and mature brain. Our study also sheds light on the importance of exploring the possible contribution of the dysfunctions of age-dependent cilia signaling to neurodevelopmental and neurodegenerative disorders as related to the peaks of the cilia genes’ expressions across the human lifespan. 

## Data Availability

Data are available in Tables S1 and S2.

## Supplementary Materials

The following are available online at https://www.mdpi.com/article/10.3390/ijms221910387/s1.
