# Supplementary material for: Dynamic Changes of Brain Cilia Transcriptomes across the Human Lifespan
Source: Int J Mol Sci. 2021 Sep 27;22(19):10387. doi: 10.3390/ijms221910387 (PMC8509004; doi:10.3390/ijms221910387)
Supplement: Supplementary file 1 [file ijms-22-10387-s001.zip › Chen et al- Supplementary Material Titles and Legends.pdf]

Supplementary Figures

Figure S1. Heatmap representations of pairwise correlations between expressions of each cilia genes pairs

a-p Brain region-specific heatmap representations of pairwise Pearson's correlations between expressions of each pair of cilia genes across age represented as a matrix in the 16 human brain regions. The Pearson correlation coefficients were calculated for each pair of cilia genes across four months to 40 years. Dark red indicates high positive correlation ($r \rightarrow 1$), and dark blue indicates high negative correlation ($r \rightarrow -1$), and white indicates no correlation ($r \cong 0$).

Figure S2. Sub-cilia localization of the genes that exhibited age-differential gene expression in the 16 brain regions

a-p. Sub-cilia localization of genes that exhibited age-differentially expression in anterior (a) ACC, (b) AMY, (c) CER, (d) DLPC, (e) Hipp, (f) ITC, (g) MTh, (h) OFC, (i) PAC, (j) PMC, (k) PPC, (l) PSSC, (m) PSTC, (n) PVC, (o) STR, and (p) VLPC. Upregulated and downregulated DEGAs are in orange and blue, respectively.

Supplementary Tables

Supplementary Table 1 (Table S1): List of cilia genes with their expressions in 16 human brain regions of 42 individuals ranging from 4 months to 40 years old.

Supplementary Table 2 (Table S2): Cilia gene transcripts that fit in the linear regression model ($P < 0.05$) in 16 human brain regions together with the linear regression coefficient and P-values.
