# Supplementary figures and images for: Dynamic Changes of Brain Cilia Transcriptomes across the Human Lifespan
Source: Int J Mol Sci. 2021 Sep 27;22(19):10387. doi: 10.3390/ijms221910387 (PMC8509004; doi:10.3390/ijms221910387)

# Supplementary Figures

Figure S1

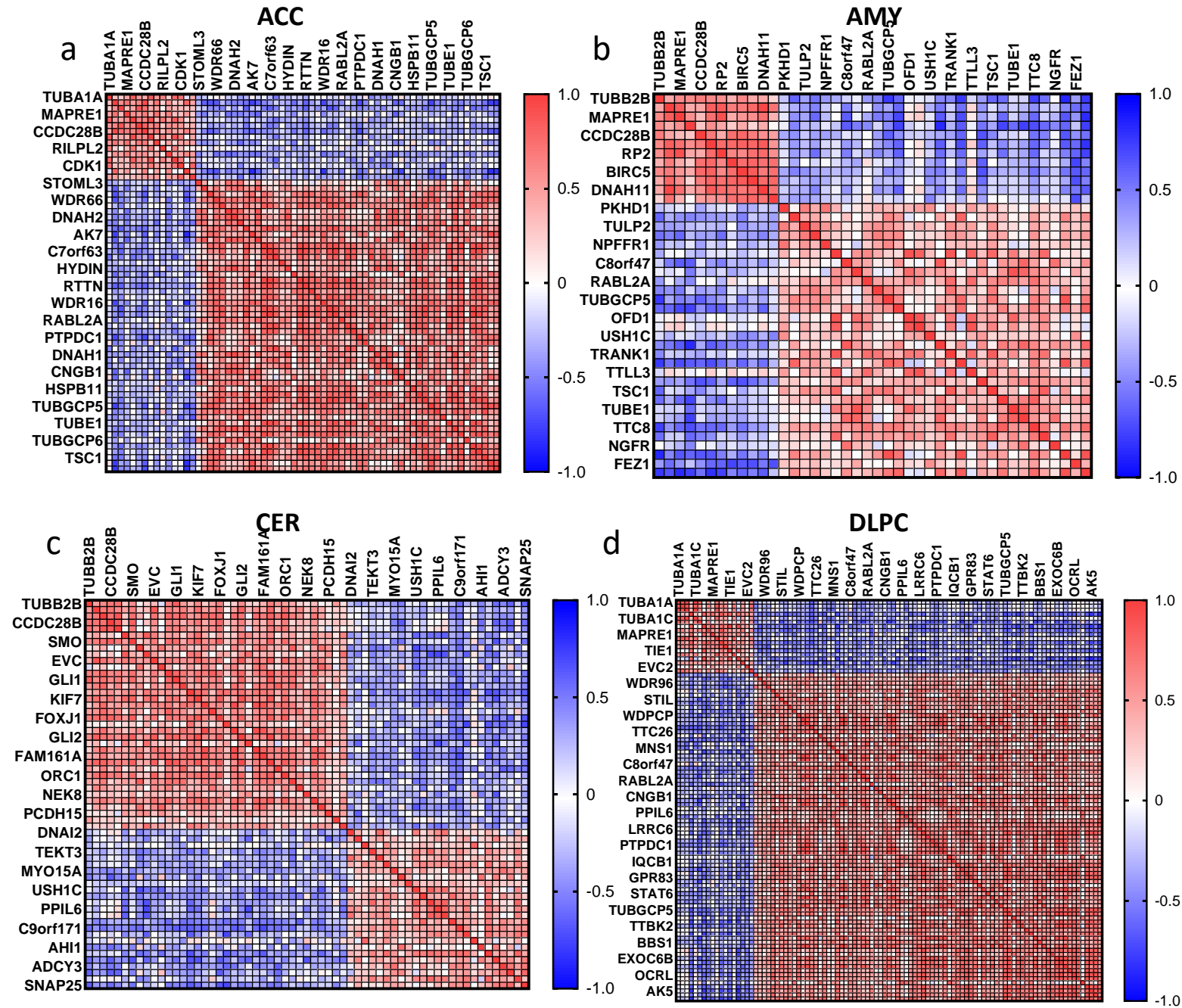

Figure S1

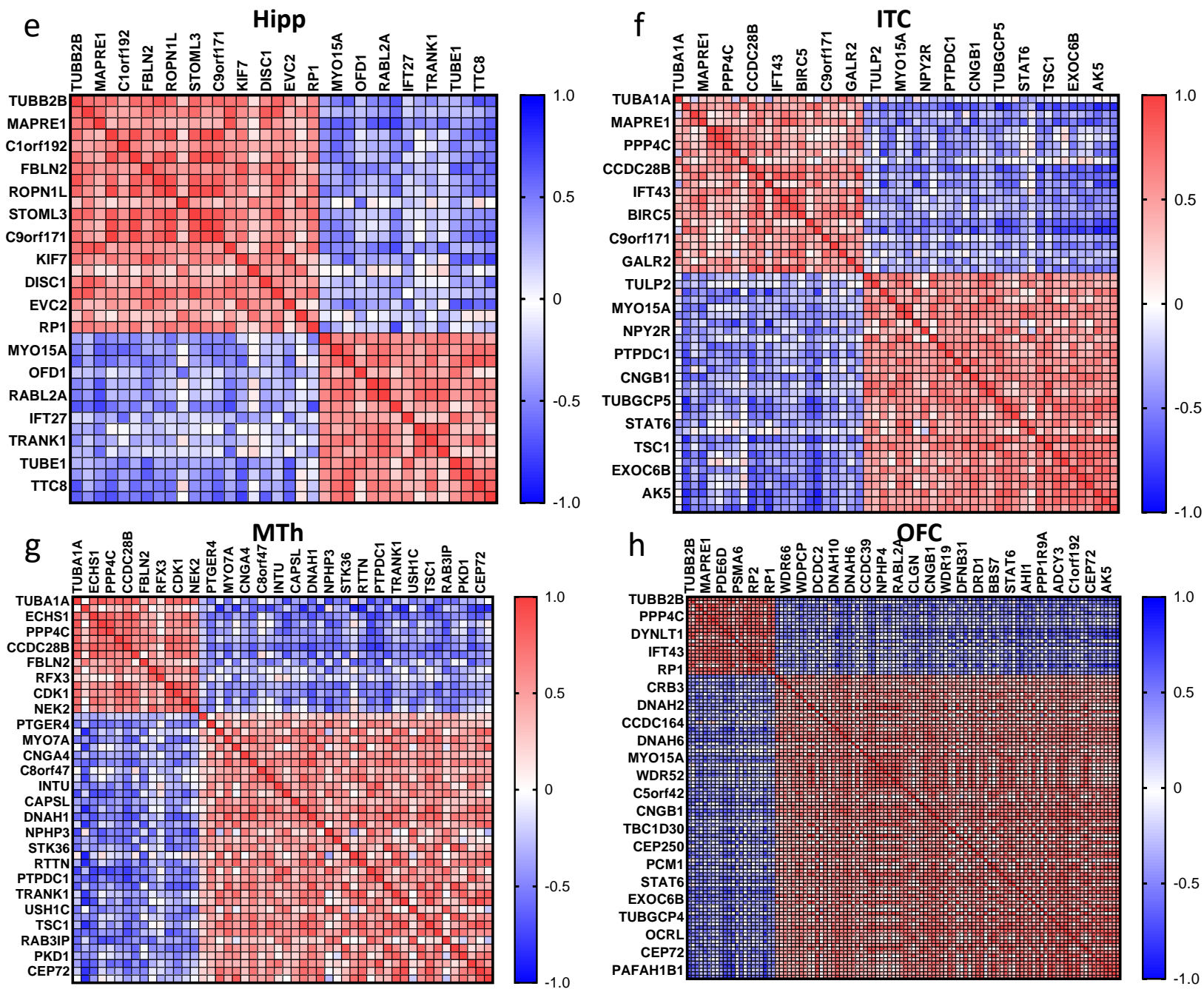

Figure S1

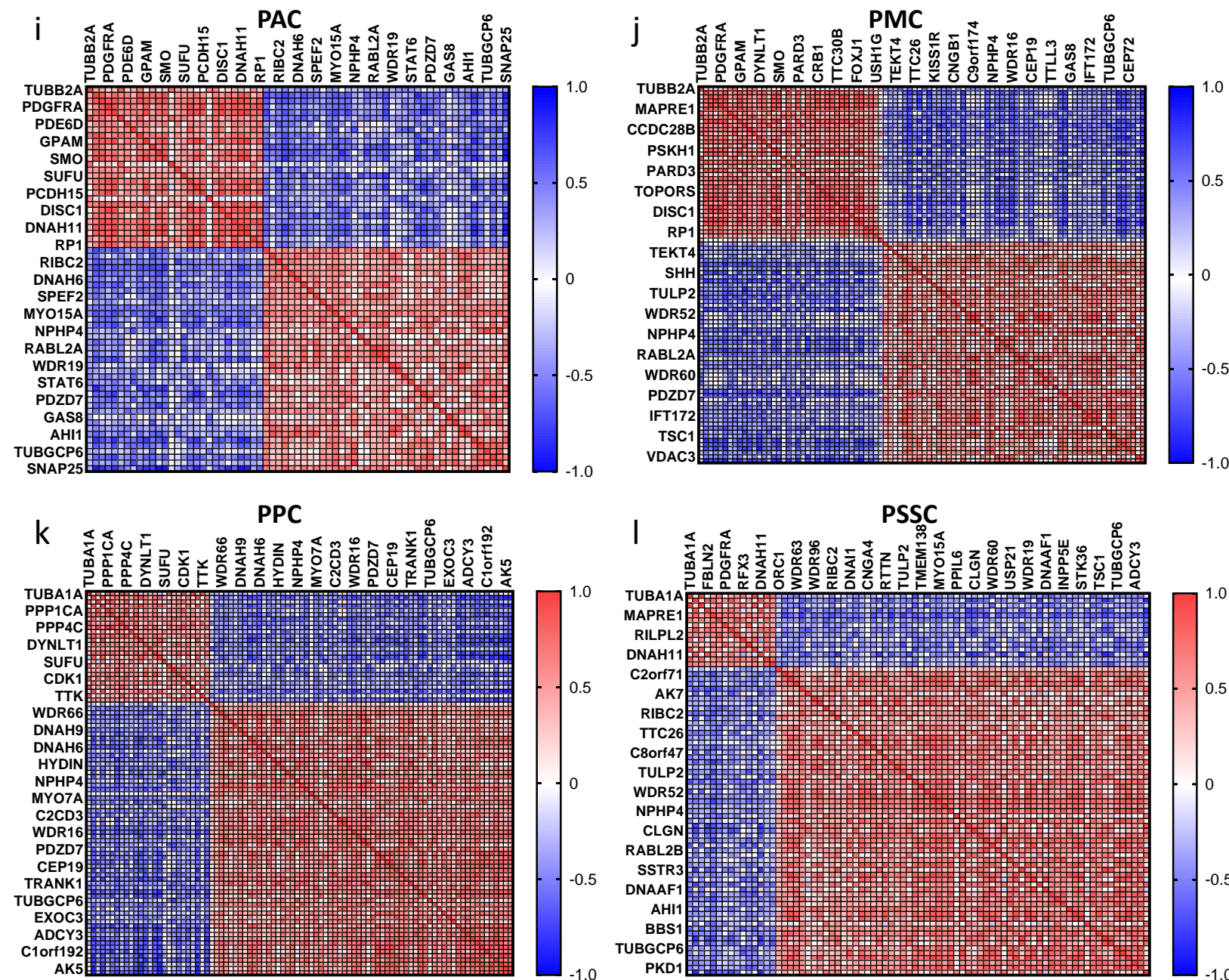

Figure S1

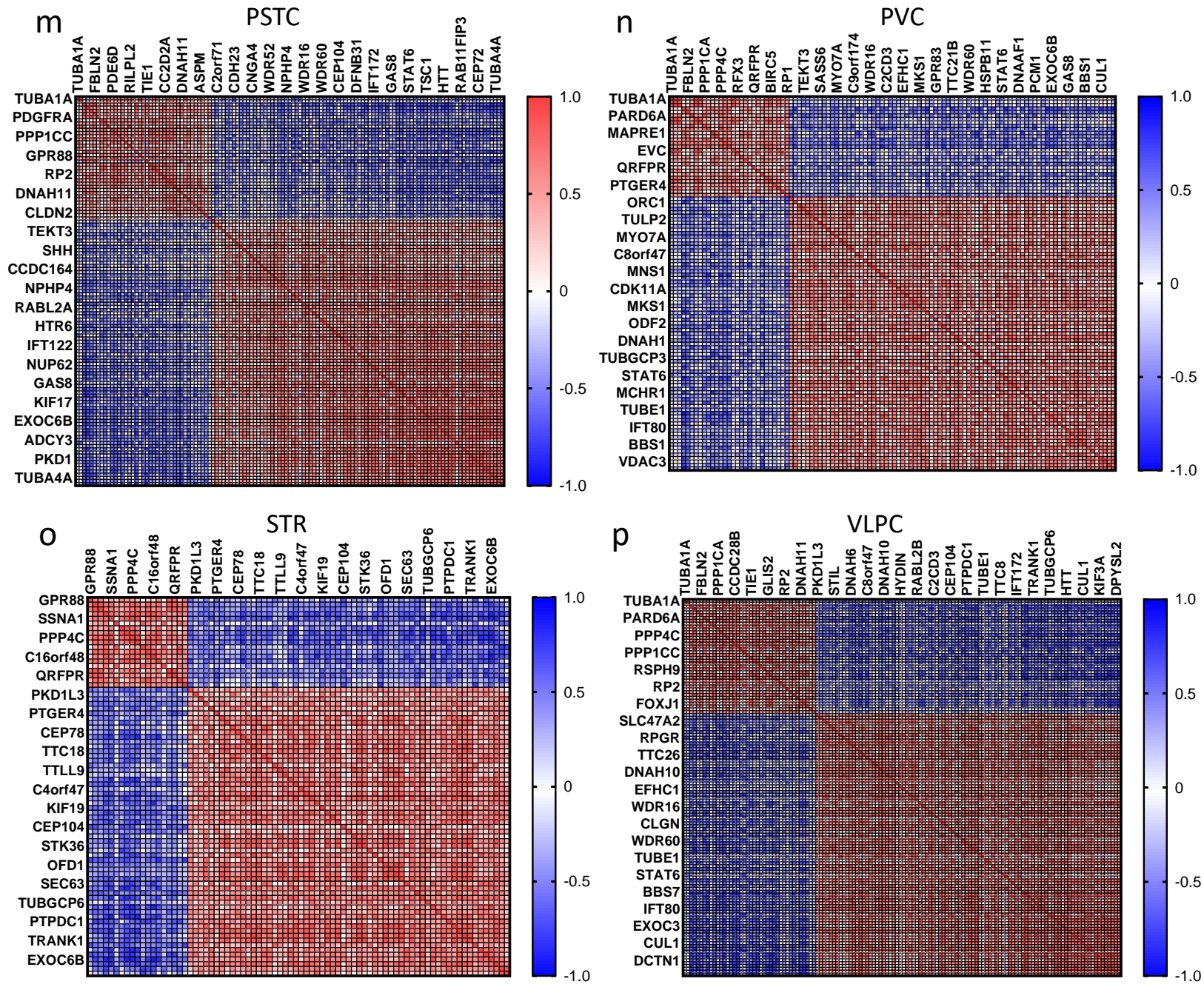

Figure S2

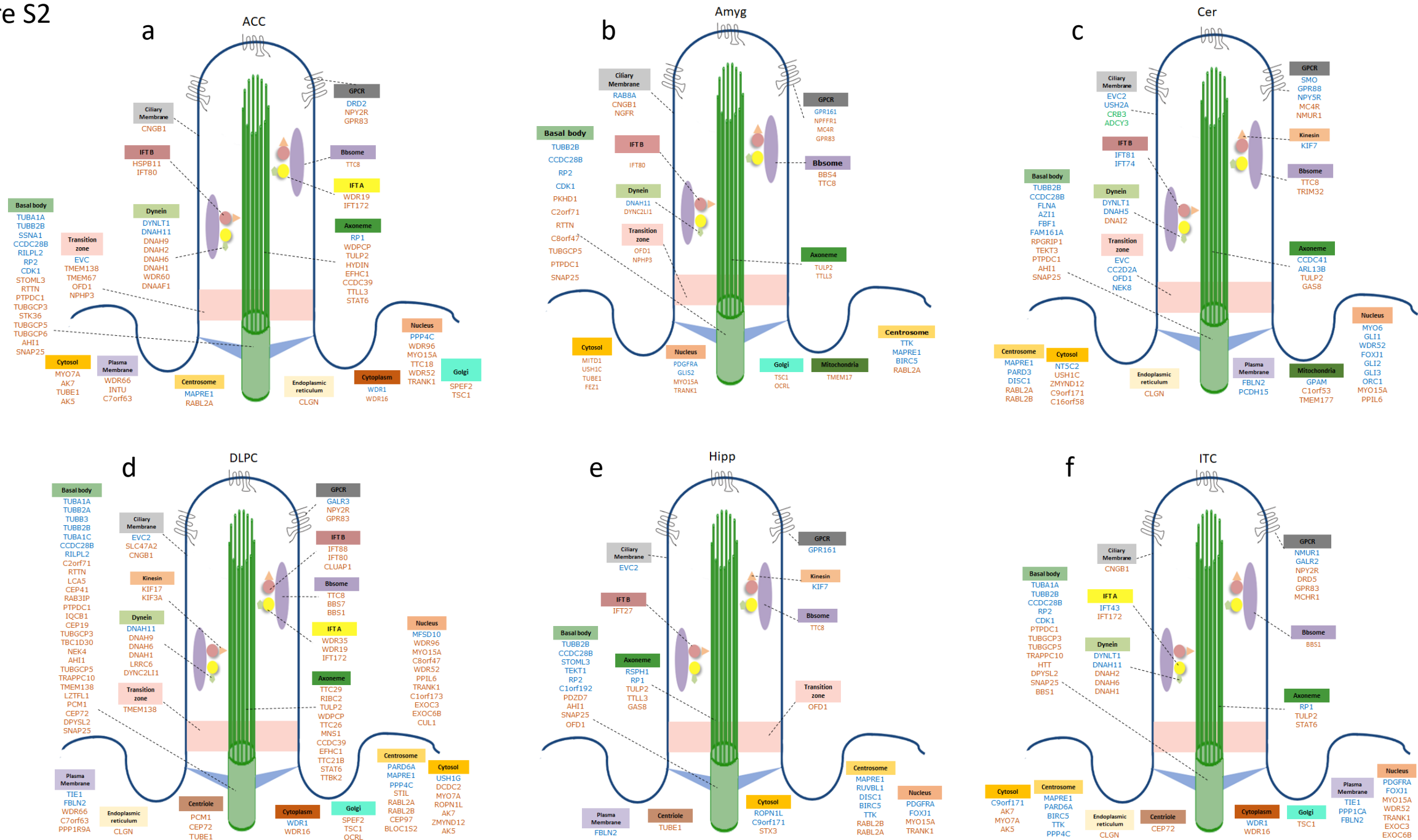

Figure S2

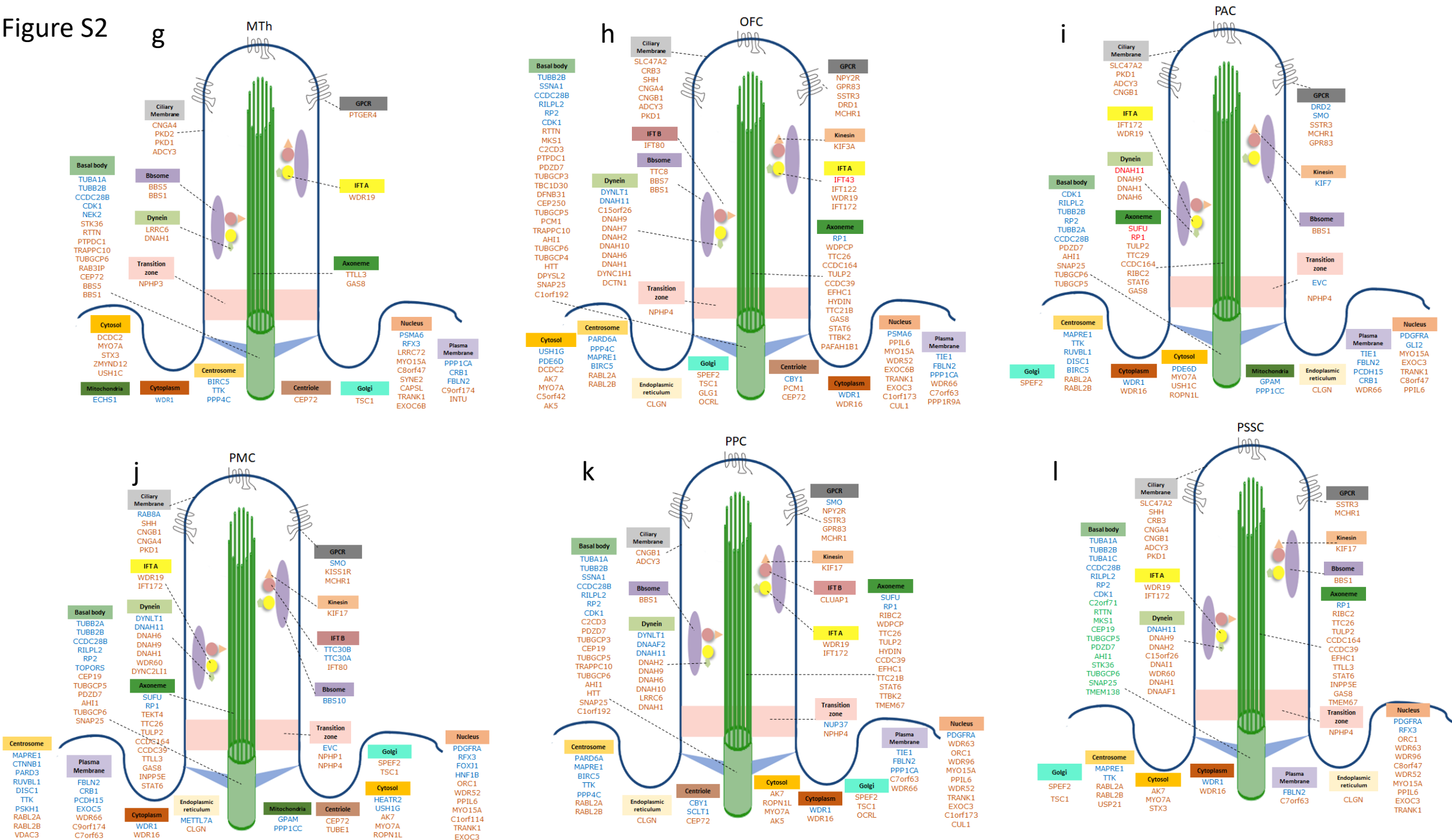

Figure S2

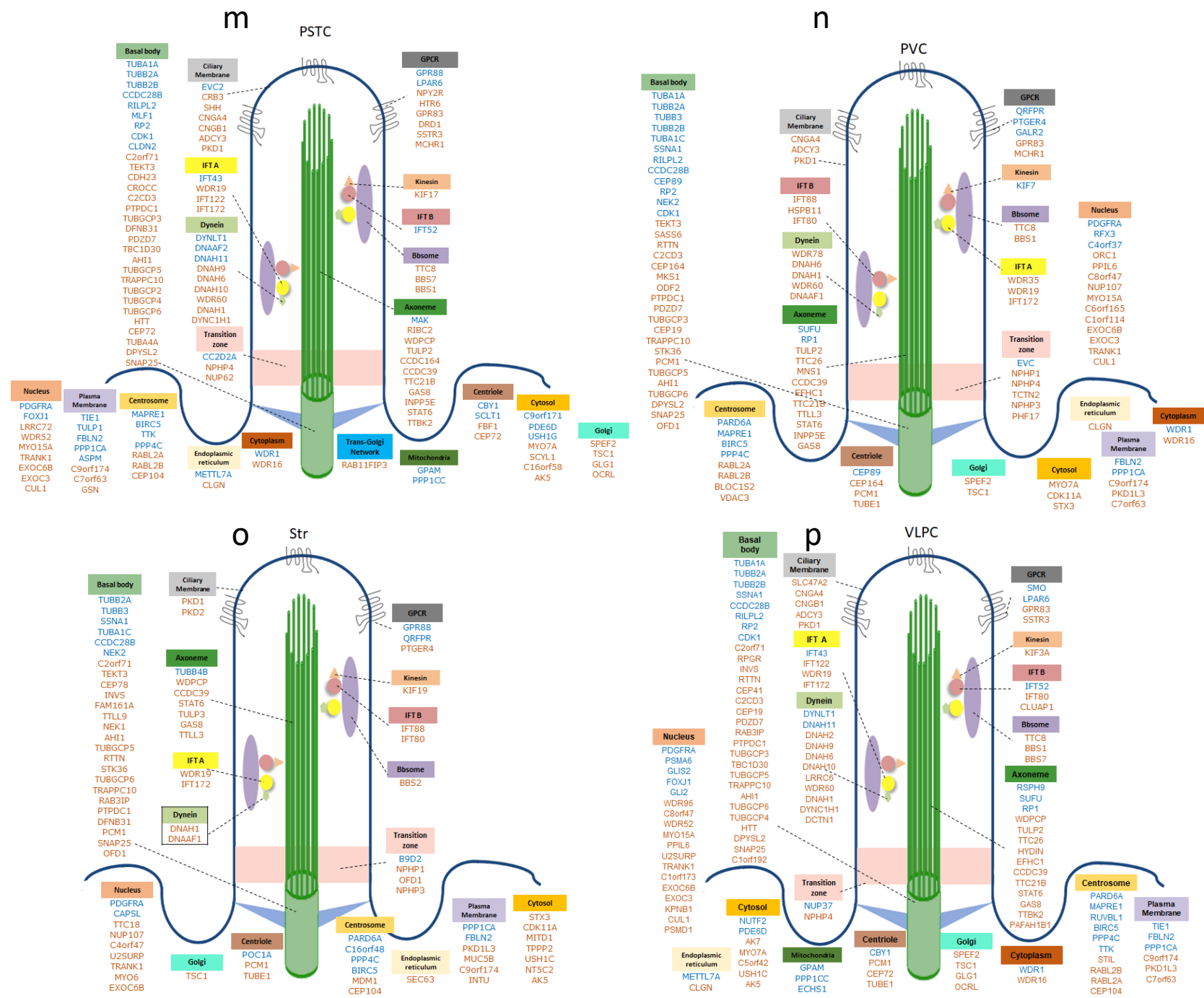

Supplement: Supplementary file 1 [file ijms-22-10387-s001.zip › Chen et al- Revised Supplemental Figures 2nd revision.pdf]
